# Digital Dynamic 3D Monitoring of Lower Incisors Intrusion in Lingual Orthodontics

**DOI:** 10.2174/1874210601812010104

**Published:** 2018-01-31

**Authors:** Elia Kodjo Chardey, Rosamaria Fastuca, Matteo Beretta, Alberto Di Blasio, Nicolò Vercellini, Alberto Caprioglio, Piero Antonio Zecca, Aldo Macchi

**Affiliations:** 1 Department of Surgical and Morphological Sciences, University of Insubria, Varese, Italy; 2 Department of Biomedical Sciences, Dentistry and Morphological and Functional Imaging, University of Messina, Messina, Italy; 3 Department of Biotechnological, Biomedical and Translational Sciences, Section of Orthodontics, University of Parma, Parma, Italy; 4 Department of Medicine and Surgery, University of Insubria, Varese, Italy; 5 Department of Biotechnology and Life Sciences, University of Insubria, Varese, Italy

**Keywords:** Lower intrusion, Root position, Lingual orthodontics, Digital setup, 3D imaging, Deepbite, Anterior crowding

## Abstract

**Objective:**

The aim of the present study is to propose a 3-dimensional evaluation of lower intrusion obtained with lingual orthodontics considering not only the crowns but also dental roots.

**Methods:**

9 adult patients underwent fixed lingual orthodontic treatment with i-TTя lingual brackets system for the correction of crowding in the lower arch associated with a deep overbite. Initial records, consisting of photos, CBCTs and intraoral scans were collected. Threshold segmentation of the CBCT was performed to generate a three-dimensional virtual model of each the teeth of the lower arch, superimposed with the crown of the same teeth obtained by intraoral scan models to generate a complete set of digital composite lower arch The same procedure was performed to monitor one key step of the i-TT_Я_ technique consisting in lower incisors intrusion (T2). T1-T2 three-dimensional superimposition and color displacement maps were generated to measure and evaluate the movements obtained at the lower arch.

**Results:**

The root displacement of the incisors during their intrusion in the early stage was totally “bone-safe” in the 88.9% (8 of 9) of the cases observed. No significant extrusion of the premolars used as anchorage unit was measured.

**Conclusion:**

This method has proved to be an accurate and reliable approach to dynamically visualize the 3-dimensional positions of the teeth, including their roots, with no additional radiation for in-progress treatment monitoring. The 3-dimensional evaluation showed that the employed lingual appliance allowed to obtain significant lower incisors intrusion with negligible undesired extrusion of premolars employed as anchorage teeth.

## 1 INTRODUCTION

In Orthodontics different appliances are used to move teeth from a malocclusion to a functional and stable occlusion with the teeth in their proper relationships as well as in harmony with the maxillofacial hard and soft tissues. The six fundamental keys that describe teeth locations in three-dimensional (3D) space defined by Andrews [1-4] were suggested for successful orthodontic treatment outcomes. However, because of inaccuracies in bracket positioning during the initial bonding process and variations in tooth anatomies and bracket designs, Andrews' six keys are difficult to achieve for even experienced orthodontists. Furthermore, because four of Andrews’ keys (mesiodistal, faciolingual, occlusal gingival position and axial rotation) focused solely on the crown and not on the entire tooth, root position at the end of treatment might be compromised [5-8].

One of the most frequent problem to solve in our orthodontic treatment is to find a proper mechanics for lower incisors intrusion in adult patients, considering the risk of unwanted side effects, such as buccal tipping of incisors associated to distal and/or buccal tipping of the anchorage units, usually represented by the posterior teeth, resulting in possible periodontal problems [9]. Recently, lingual fixed appliances have been described as effective for reaching lower incisors intrusion minimizing side effects [10-16].

But is it possible to do that or even prevent side effects by accurately in progress monitoring the treatment and not only at the beginning or at the end? Traditionally, monitoring and finalizing the root positions has been performed using panoramic X-rays at the initial, progress and finishing stages of orthodontic treatment. Anyway, many studies [17-19] have indicated that panoramic X-rays do not accurately reflect the true root positions, especially of the canines and first premolars, as a result of distortions mainly due to the X-ray beams, not being orthogonal to the target teeth. In recent years, the development and use of Cone Beam Computed Tomography (CBCT) has allowed for accurate visualization of the roots of teeth in 3 dimensions. However, a CBCT scan uses significantly more radiations than does a panoramic radiograph, so multiple CBCT scans would not be suggested clinically. An imaging technique that can be performed more times during orthodontic treatment without radiation is a digital intraoral surface scan. This technique can accurately display crowns with high resolution, but it cannot show the roots. Since neither CBCT nor digital intraoral surface scans can safely and accurately visualize root positions at different stages of orthodontic treatment, root tracking might be possible with a combination of these two imaging techniques.

Lee *et al*. [20, 21] recently showed the validity of a new method to accurately visualize root positions comparing the beginning and the end in vestibular orthodontic treatment: a digital intraoral surface scan combined with a pre-treatment CBCT scan, resulting in a safe and accurate root position assessment in three dimensions throughout orthodontic treatment. However, this study didn’t give us an idea of the possibility of monitoring orthodontic treatment *in progress* and did not show how the presence of the brackets (either vestibular or lingual) could affect the efficiency of the method suggested.

An important objective of orthodontic treatment is to achieve proper and stable tooth positions that involves not only the crowns, but also their roots. However, the current methods of clinically monitoring root alignment are unreliable and inaccurate. According to our previous study [16], that shows the efficiency on adult patients of an original mechanics for lower incisors intrusion using lingual appliances, and starting from the method proposed by Lee *et al*. [20, 21], the aim of the present study was to propose a three-dimensional and dynamic evaluation of lower incisors intrusion, obtained with i-TTя lingual system and to measure how effective it is in term of respect of the alveolar bone and periodontal tissues.

## 2 MATERIALS AND METHODS

### 2.1 Population and Study Design

This study followed a prospective longitudinal design and enrolled subjects seeking orthodontic treatment for crowding correction. A signed informed consent for releasing diagnostic records for scientific purposes was obtained from all the patients. The protocol was reviewed and approved by the Ethic Committee (Approval no. 1532) and procedures followed adhered to the World Medical Association Declaration of Helsinki. Briefly, the following criteria were applied: i) adult patients with complete permanent dentition ii) good general health according to medical history and clinical judgment [22-24]; iii) patients seeking orthodontic treatment specifically enrolled for lingual orthodontics; iv) Class I malocclusion clinically evaluated; iv) a deep bite malocclusion needing a certain amount of intrusion of the lower incisors for its correction. Exclusion criteria were as follows: severe crowding, dental or craniofacial anomalies, extraction cases and patients affected by periodontal disease.

**Fig. (1):**
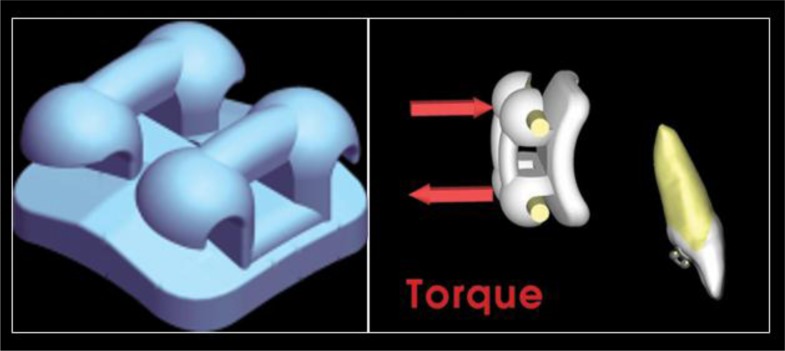
i-TTя bracket design.

**Fig. (2):**
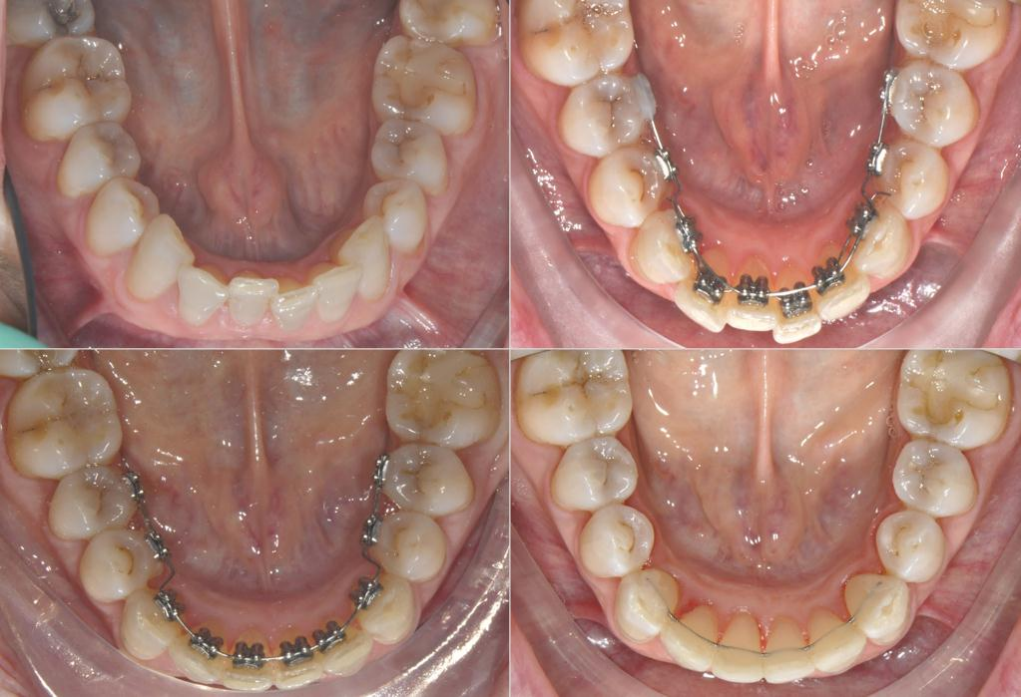
Example of a clinical case. Occlusal views of the lower arch through various steps of the treatment.

**Fig.(3):**
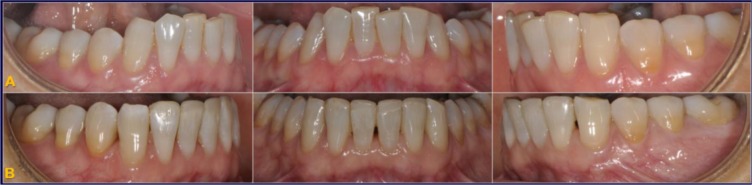
(A-B). Example of a clinical case. Levelling and alignment of the lower arch achieved due to controlled and modulated intrusion of the anterior segment. A) Before treatment B) After lower incisors intrusion.

This research was designed as a pilot study. The final sample analyzed was the following: 9 patients (5 women and 4 men), with a mean age of 29.04 ± 0.8 years old. All the patients were treated by the same experienced orthodontist (M.B.). For all the patients, i-TTя lingual brackets (Rocky Mountain Orthodontics, Denver, USA, (Fig. **1**) were bonded from the lower right second premolar to the lower left second premolar (Figs. **2**, **3**) [15, 16].

Initial records including CBCT and intraoral surface scans were taken. Each patient was subjected to a CBCT (Newtom Giano, CEFLA S. C., CEFLA DENTAL GROUP, Imola, BO, Italy; 90 kV, 10 mA, 18-seconds scan time). CBCT scans were taken prior to be enrolled for orthodontic treatment, for the diagnostic set [25-27]. A 3Shape TRIOS® intraoral scanner (3Shape A/S, Copenhagen K, Denmark) was used to digitally scan the crowns of the teeth of each patient before treatment (T1) and at the end of the lower incisors intrusion stage (T2, 0.67 ± 0.15 year).

### 2.2 Image Processing

DICOM files of each patient (obtained from the CBCT scan) were imported into Mimics (version 17.0; Materialise, Leuven, Belgium). A threshold segmentation, individual for each single patient analyzed, was applied resulting in the generation of 3D virtual surface models of the mandibular dental arch, including crowns and roots of all the teeth.

After the creation of the mask, each tooth was isolated from its respective arch export as an independent binary STL file to create a database of digital teeth for every single patient of the sample.

**Fig.(4):**
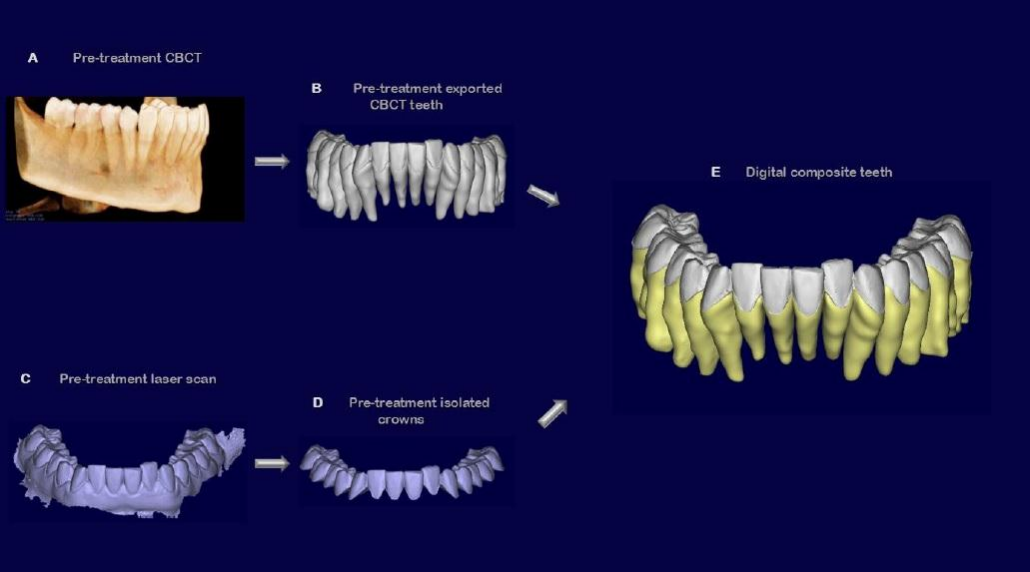
Generation of individual digital teeth. Threshold segmentation of the CBCT was performed to generate a three-dimensional virtual model of each teeth of the lower arch, superimposed with the crown of the same teeth obtained by intraoral surface scan models to generate a complete set of digital composite lower arch.

A 3 Shape intraoral scanner (3 Shape A/S, Copenhagen K, Denmark) was used to digitally scan the crowns of the teeth of each patient, resulting in stl files of the mandibular and maxillary arches in high resolution. The stl files of these arches were imported into the software Geomagic Studio 2014^®^ (version 3.0.1781, 3D Systems), in which the crowns of the intraoral scans were also individually isolated into their own crown parts for single-crown manipulation. Since intraoral scans do not capture the roots, they were combined with the teeth previously obtained by the cbct, after oriented all of them in the same reference system. The high-resolution crowns from the intraoral scan were joined with the teeth from the cbct scan, creating individual digital “composite teeth” (Fig. **4**).

**Fig.(5):**
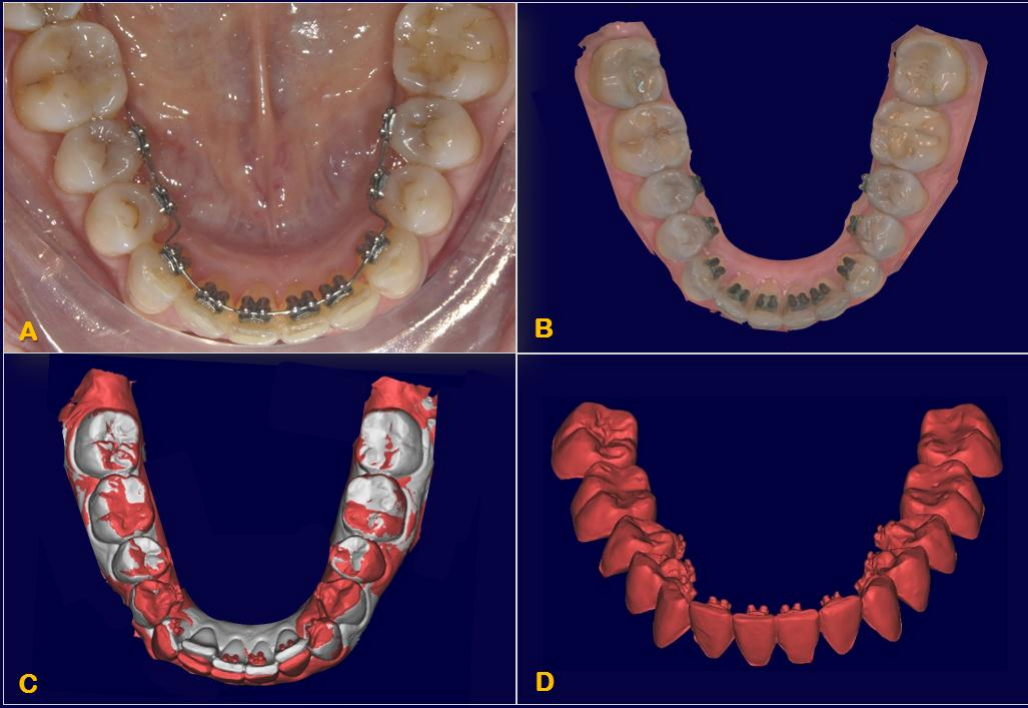
Generation of T1 individual digital crowns after taking laser scansion at the end of the intrusion stage. A) Clinical view B) Digital 3D laser scansion C) T1 scansion aligned to T0 at the level of lower molars, not banded during the treatment D) Isolated T1 crowns.

**Fig.(6):**
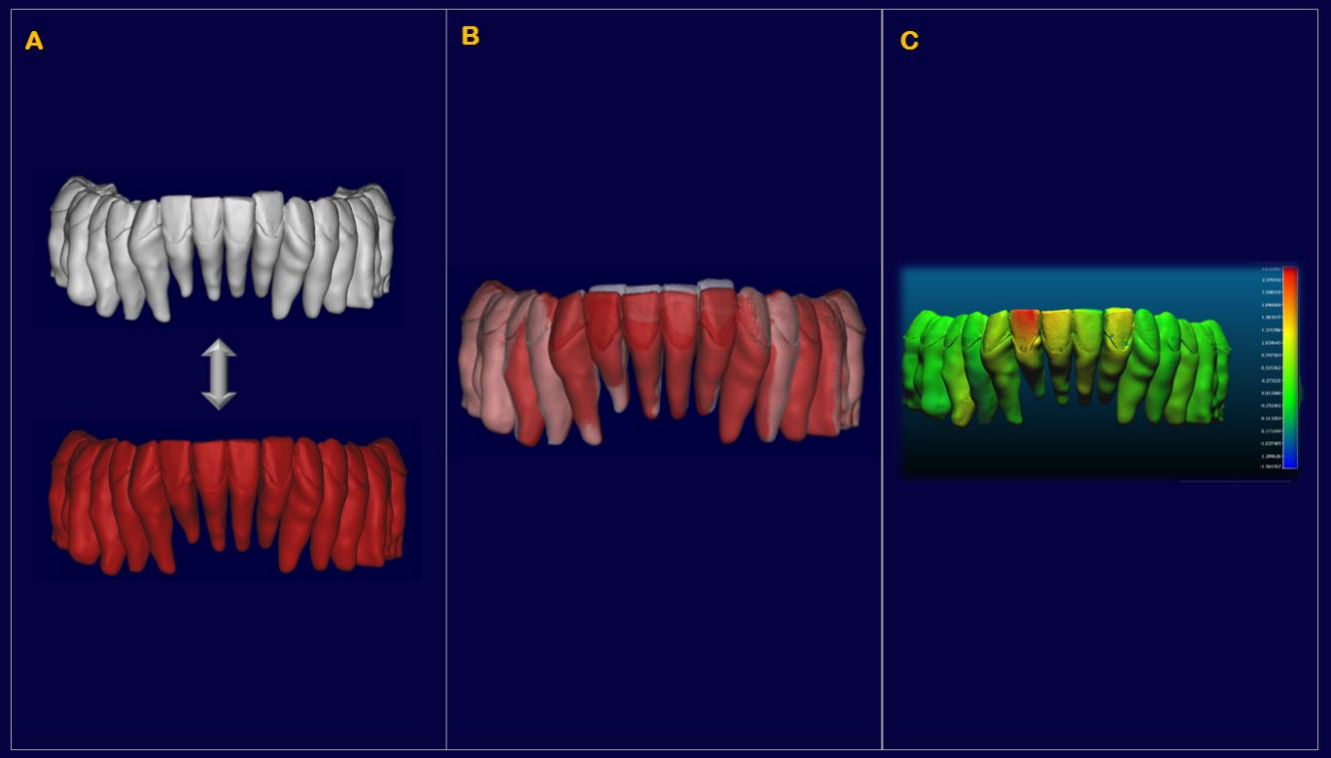
Generation of direct superimposition and colour maps.

At interval T2, a new set of individual isolated crowns was created after the alignment of the T2 laser scansion with the T1 one, performed at the level of the lower molars not included in the active treatment (Fig. **5**). Then, every single T1 tooth was realigned independently on the new T2 high-resolution crown to create a new set of T2 teeth. At the end of the process, each “in-progress*”* tooth was currently superimposed with the initial tooth position onto the software Cloud Compare^®^ (version 2.6.2) to create a color map that measured the displacements between the T1 and the T2 isolated teeth, especially in term of anterior intrusion and control of the bite (Fig. **6**).

### 2.3 Method Error

The same trained operator (E.C.) performed and repeated all the procedure three months later. A color map was created to state the displacement between the first and the second set of digital T1 and T2 isolated teeth. Systematic and random errors were calculated comparing the first and the second set with paired t-test and Dahlberg’s formula [28], at a significance level of *P* < 0.05. All measurement error coefficients were found to be adequate for appropriate reproducibility of the study.

## 3 RESULTS

For the evaluation of our sample we relied on a qualitative comparison, obtained by direct superimpositions and by the creation of color maps.

Direct superimpositions between each single pre-treatment and progress intraoral scan crowns was verified to be accurate through color maps. The color maps showed a displacement of 0.097 ± 0.385 mm for the mandibular teeth, with a maximum of 0.184 mm.

The indirect superimposition results for the pre-treatment and progress composite mandibular teeth of all the patients were very similar. The patients were numbered from 1 to 9. Qualitative assessment of dental changes was conducted using a semitransparent overlay of the superimpositions and an iterative closest point measurement from color maps. For structures that were obstructed from view, a mesh transparency for T2 allowed for better visualization of the superimpositions. All treated patients, with no exception, demonstrated a certain amount of lower incisor intrusion. Three subjects Figs. (**7a** and **8a**; case 3, 4 e 5) displayed the highest amount of intrusion, probably in relation to the presence of more crowding and incisor extrusion to be correct at the beginning of the treatment. Five patients showed buccal inclination of the incisal edge (Figs. **7b** and **8b**); case 3, 4, 5, 6 e 8). No significant extrusion of the premolars used as anchorage unit was showed (Figs. **7c**) and **8c**). Furthermore, the inter-premolar diameter remained stable, suggesting that the functional anchorage used during the treatment was reliable.

Figs. (**8a**-**c**) shows the composite of the individual color maps, demonstrating the global changes computed with the iterative closest point algorithms. Although the mandibular teeth positional changes in the treated patients showed statistical differences comparing T1 with T2, considerable variations in magnitude and direction of these dental changes were seen when examining the color maps of each subject (Fig. **7**).

## 4 DISCUSSION

Stability, correct function and esthetics are all fundamental goals for a successful orthodontic treatment. A proper placement not only of the crowns but also of the roots is required to obtain good results. Nowadays, the majority of the focus in orthodontic practice is on crown position rather than root position, because roots are not directly involved with esthetic and occlusal contacts [8].

Nevertheless researchers have found that proper placement of roots after orthodontic treatment cannot completely reduce the risk of future relapse and that the proper occlusion in each patient changes among the different stage of life, but it is reasonable to say that a proper position of the roots in the basal bone could reduce the extent of relapse during retention [29-31].

The current standard of care suggests the use of panoramic X-rays to monitor root alignment, even though many studies have shown that panoramic X-rays do not accurately depict root positions and angulations due to their two-dimensional projection [17]. Thereby, it seems to be clear that a new approach in monitoring accurately our treatments is necessary.

For optimal assessment of the roots, a 3D representation of the teeth is necessary. This has been possible in recent years through CBCT technology, which shows dentofacial structures in a 1:1 ratio [32-34]. In addition, any distortions in the CBCT images have been found to be clinically insignificant. Thus, CBCT scans accurately display the true positions of the roots. Recently, Lee and colleagues [20, 21] proposed an innovative method for an *“in vivo”* three-dimensional evaluation of an orthodontic treatment that has the potential to accomplish that tasks, but it considered only initial and final records without any dynamic control during the treatment. In fact, this “*in-vivo”* approach was carried out by extra-oral laser scansion of poured-up study models at the end of the treatment. As it was stated by the authors *“impression are difficult to take during treatment when brackets are still bonded…Hence, it was not possible to determine whether the presence of brackets would affect the methodology”*. Therefore, it is clear that using intraoral scans to monitor root movements during treatment might give us a significant advantage. As shown in our study, the presence of brackets in the lingual surface of the tooth did not affect the reliability and the efficiency of this method when direct intraoral scansion was performed: direct superimpositions between each single *pre-treatment* and *end-of-intrusion-stage* scanned crowns was verified to be accurate through color maps. Minimal differences were found in the direct superimposition between isolated *pre-treatment* and *end-of-intrusion-stage* scanned crowns. This accuracy is imperative because this process is conducted clinically [21]. It was also demonstrated that this method allows the orthodontist to see true 3D discrepancies in root positions, unlike panoramic radiograph, which gives false information of reduced discrepancies, since a 3D relationship is projected onto a 2-dimensional image.

However there were some limits in this study design, as there were some areas with complex anatomy were still difficult to segment [35, 36]. This can lead the operator to add or subtract tooth structure and cause these discrepancies, especially on the occlusal surface. The operator also may cause further errors during manual adjustment of segmentation because the operator's human vision and visual discrimination of crown, root, bone, and air are subject to a host of factors, such as lighting conditions, fatigue, gray-scale ability, and visual acuity [37]. A small amount of wrapping and smoothing were also applied to optimize the 3D surface model of the composite teeth, with the only but significant exception of the crowns for keeping a high level of accuracy: in fact, with these functions, some small changes on tooth morphology may have been introduced. Furthermore, a low CBCT image quality could affect the discrimination between densities while performing threshold segmentation [38].

The aim of our research was a three-dimensional dynamic monitoring of lower incisor intrusion mechanics obtained using i-TTя lingual bracket system in adult patients. Orthodontic treatment in adult with lower crowding and an increased overbite represents one of the main challenge both in term of efficiency and in term of long term stability: assessing a proper root position in respect of the alveolar bone’s limits is a fundamental objective to achieve. It has been suggested that in general a combination of molar extrusion and incisor intrusion occurs with the lingual appliances as a result of use of anterior bite planes that cause posterior disclusion [39-42]: the lingual system proposed is based on occlusal anchorage in order to limit posterior extrusion and excessive bite opening to make lower anterior intrusion more efficient and functional (Fig. **3**). In fact, although bite opening could be favorable in low angle brachifacial patterns, the same may induce unwanted effects that are difficult to control in mesofacial and dolichofacial ones [39]. A non-growing patient have a different skeletodental response to orthodontic bite opening than a growing patient [43]. Differently than adolescents, in adult patients, minimal extrusion of the posterior teeth will increase the mandibular plane angle, making control of the vertical plane a difficult and unpredictable task [42].

A lingual mushroom archwire was used in order to efficiently intrude the lower incisors [16]. All treated patients in our sample, with no exception, demonstrated a certain amount of lower incisor intrusion. The patients that displayed the highest amount of intrusion presented more crowding and passive incisors’ extrusion than the others at the beginning of the treatment.

**Fig. (9):**
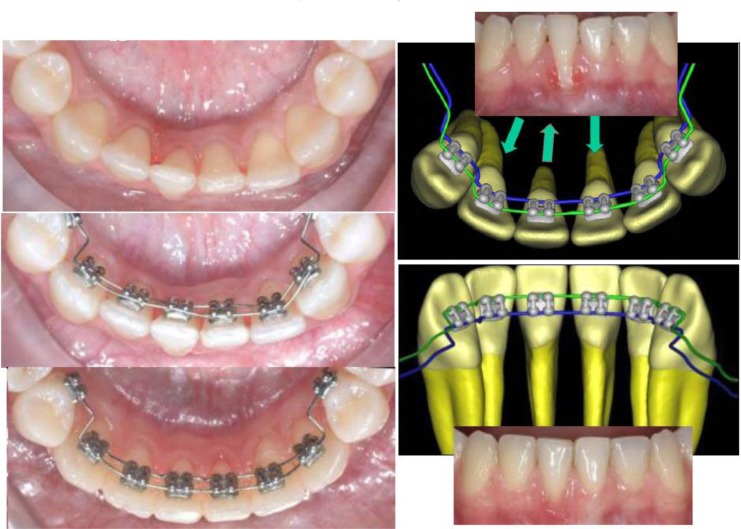
**Clinical case.** Selective root torque control in in a clinical case with gingival recession.

Five patients showed buccal inclination of the incisal edge, but the amount is negligible. Moments created with a lingual bracket system as compared with labial brackets are always smaller, with less side effects as proclination of the crown. When the bone level is reduced and the incisors are proclined, the counterclockwise moments developed with intrusive forces are more favourable than those developed with the labial brackets, because of the forces are more directed to the center of resistence in the lingual system. Moreover, the combination of two parallel and discordant forces [15] in i-TTя lingual technique allowed us a modular and efficient control not only of the vertical intrusive forces but also of the dental mesio-distal angulation, rotation and bucco-lingual inclination. So, in all the cases, during leveling and alignment stage we had a good control of the radicular torque of one or more elements we looked for when treatment objectives were established (Fig. **9**), even in that cases where the periodontal support is reduced (as case n°9). Placing the roots in its proper position in respect of periodontal support and alveolar bone is imperative to not heightened unfavorable sequelae, above all for mandibular incisors that, more frequently than maxillary ones, are the cause of limitation in treatment, because of the thinness of the alveolar housing [44, 45].

The main limitation of this method was that it is currently too time-consuming for use in a clinical setting and the sample was limited then the results are limited to the evidence of a pilot study. Considerable amounts of time and effort were necessary to perform threshold segmentations of the teeth in complex craniofacial bony structures. In addition, creating the many superimpositions that are needed is also a time-consuming process. The process of creating composite teeth requires significantly more time for the additional step of cutting out the crowns of the high-resolution intraoral scans, individually superimposing all of these crowns onto the pretreatment CBCT crowns and suturing them together. Furthermore, the alignment between T0 and T1 digital teeth was possible and accurate because performed on lower molars, not bonded and stable during the entire treatment. Further investigations are necessary to identify new stable points (outside dental arch) to perform this important step. Probably in the future, considering how digital technology is evolving fast in all the fields of dentistry [46-48], this entire process would be automatic, which could then make this methodology clinically useful in treatment planning and monitoring not only for fixed orthodontics but also for interceptive treatment [49, 50].

## CONCLUSION

- The three-dimensional evaluation showed that the employed i-TTя lingual bracket system allowed to obtain significant lower incisors intrusion with negligible undesired extrusion of premolars employed as anchorage teeth.
- This method has been showed to be an accurate and reliable approach to dynamically visualize the 3-dimensional positions of all teeth, including the roots, with no additional radiations, used not only for comparing initial and final records of a treatment, but also for *in-progress* monitoring.
- It is emphasized the fourth dimension of the diagnosis, the time factor, that represents the 4D Orthodontics concept. The limits of the proposed approach could be related to the method of thresholding based segmentation on CBCT, and further advancements in software and technology are still needed for its clinical routinely use.
- The alignment between T0 and T1 digital teeth was possible and accurate because performed on lower molars, not included in the appliance and stable during the entire treatment. More investigations are necessary to identify new stable points (outside dental arch) to perform it.

## Figures and Tables

**Fig. (7a):**
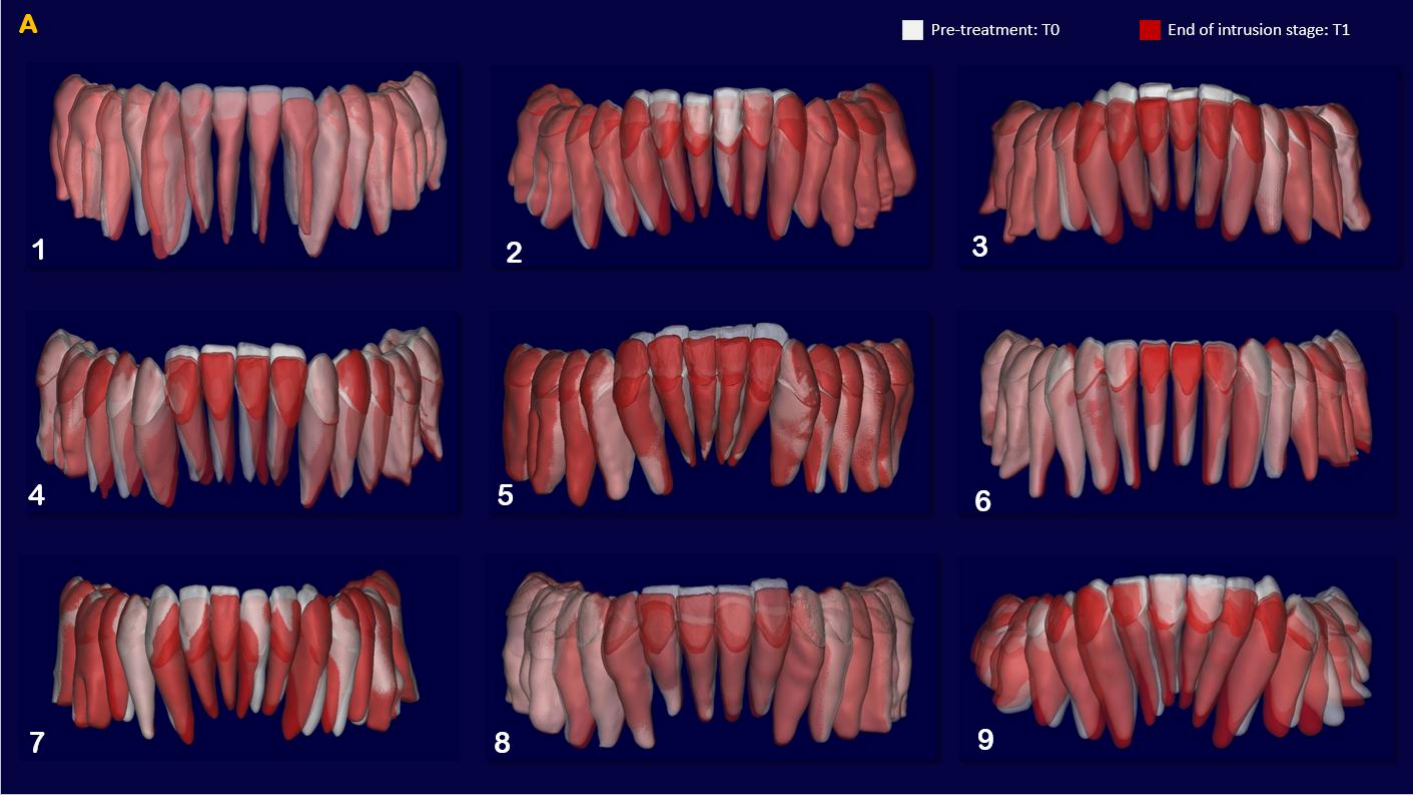
Direct superimposition between T0 and T1. Frontal view.

**Fig. (7b):**
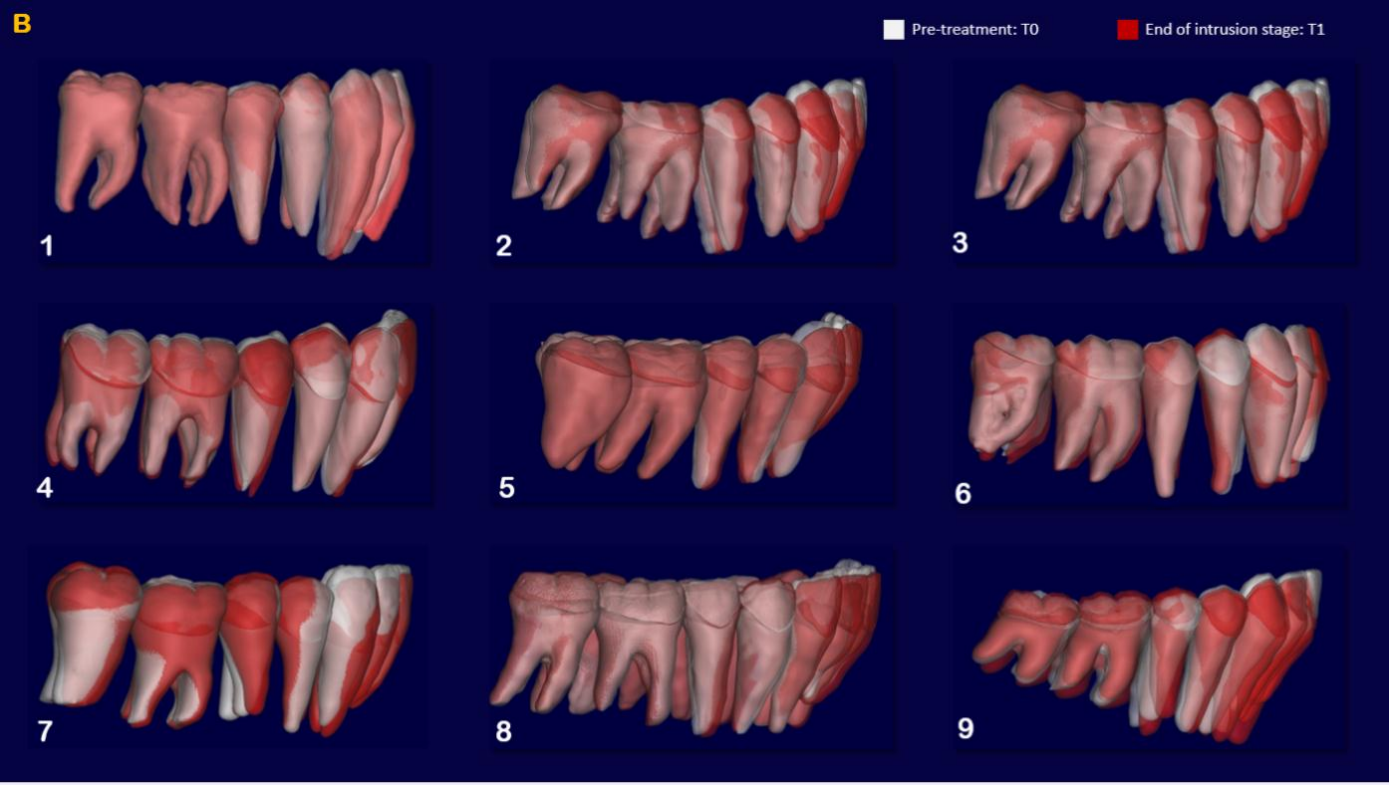
Direct superimposition between T0 and T1. *Lateral view*.

**Fig. (7c):**
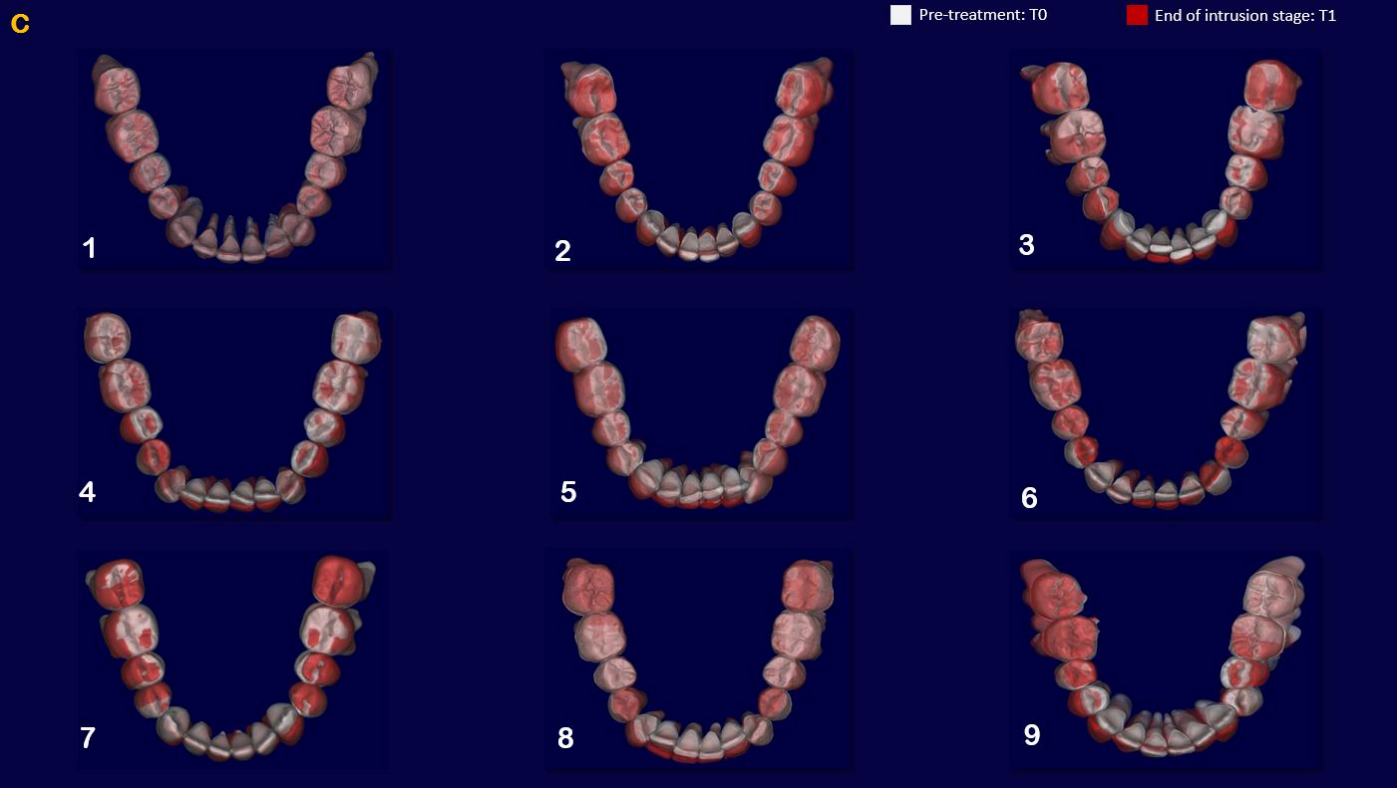
Direct superimposition between T0 and T1. Occlusal view.

**Fig. (8a):**
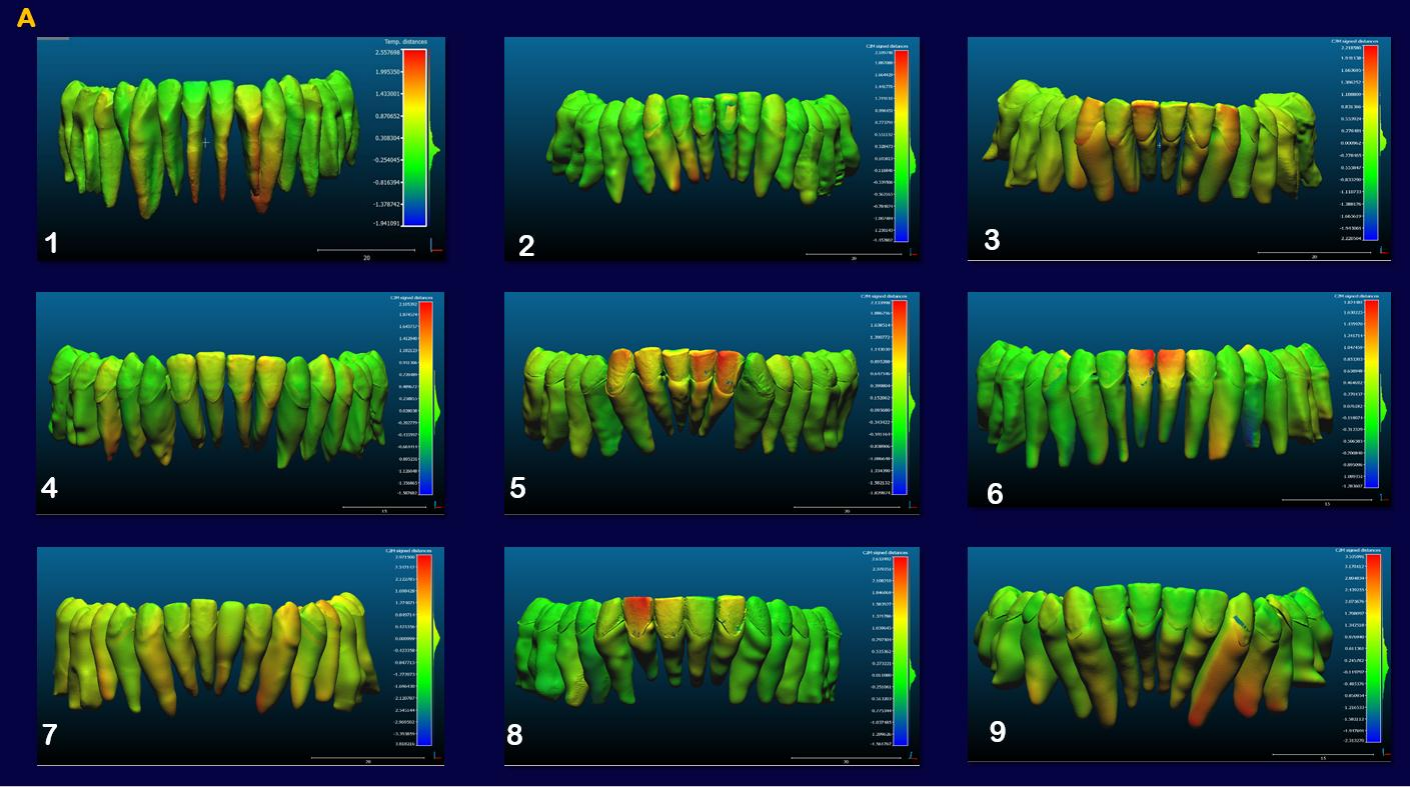
Composite of colour maps showing dental displacements between T0 and T1. Frontal view.

**Fig. (8b):**
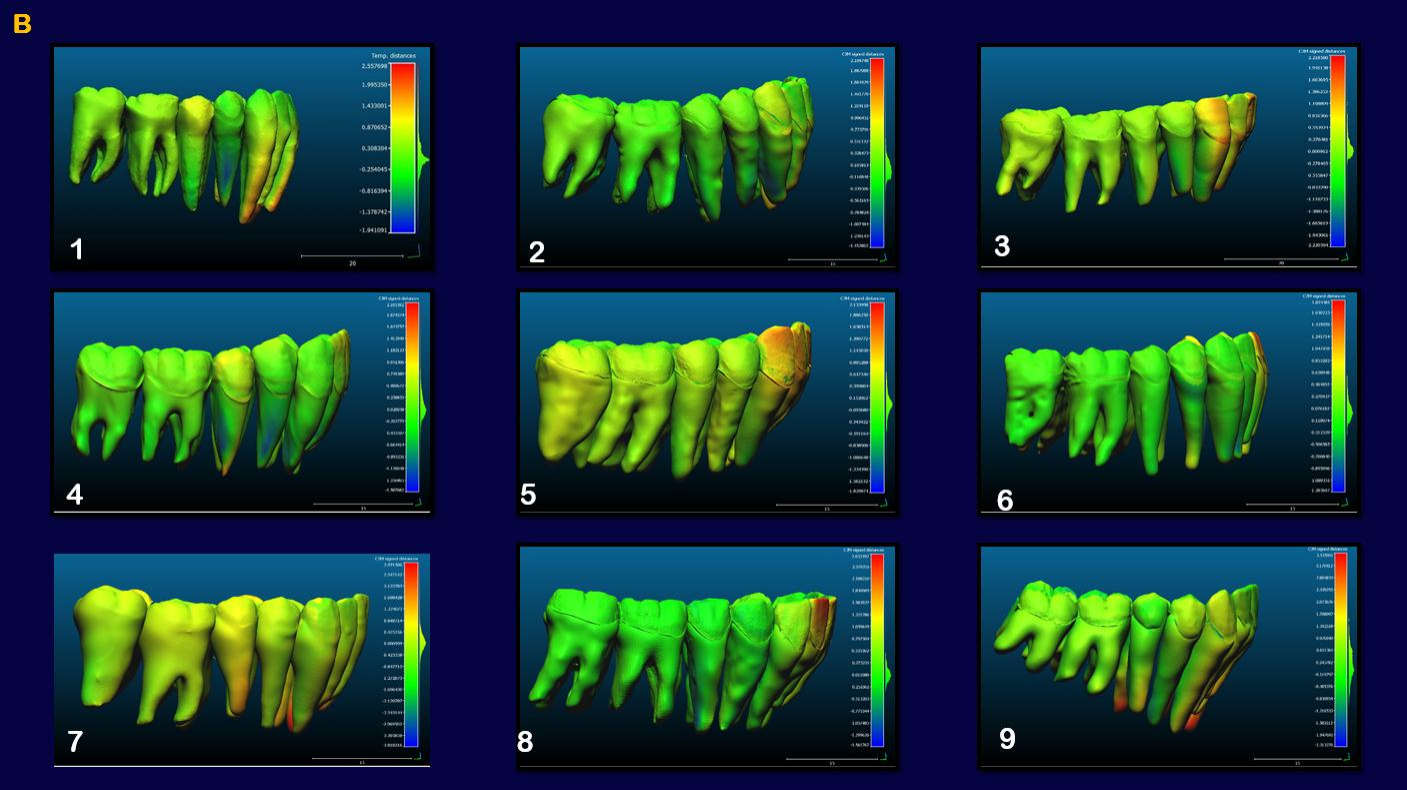
Composite of colour maps showing dental displacements between T0 and T1. Lateral view.

**Fig. (8c):**
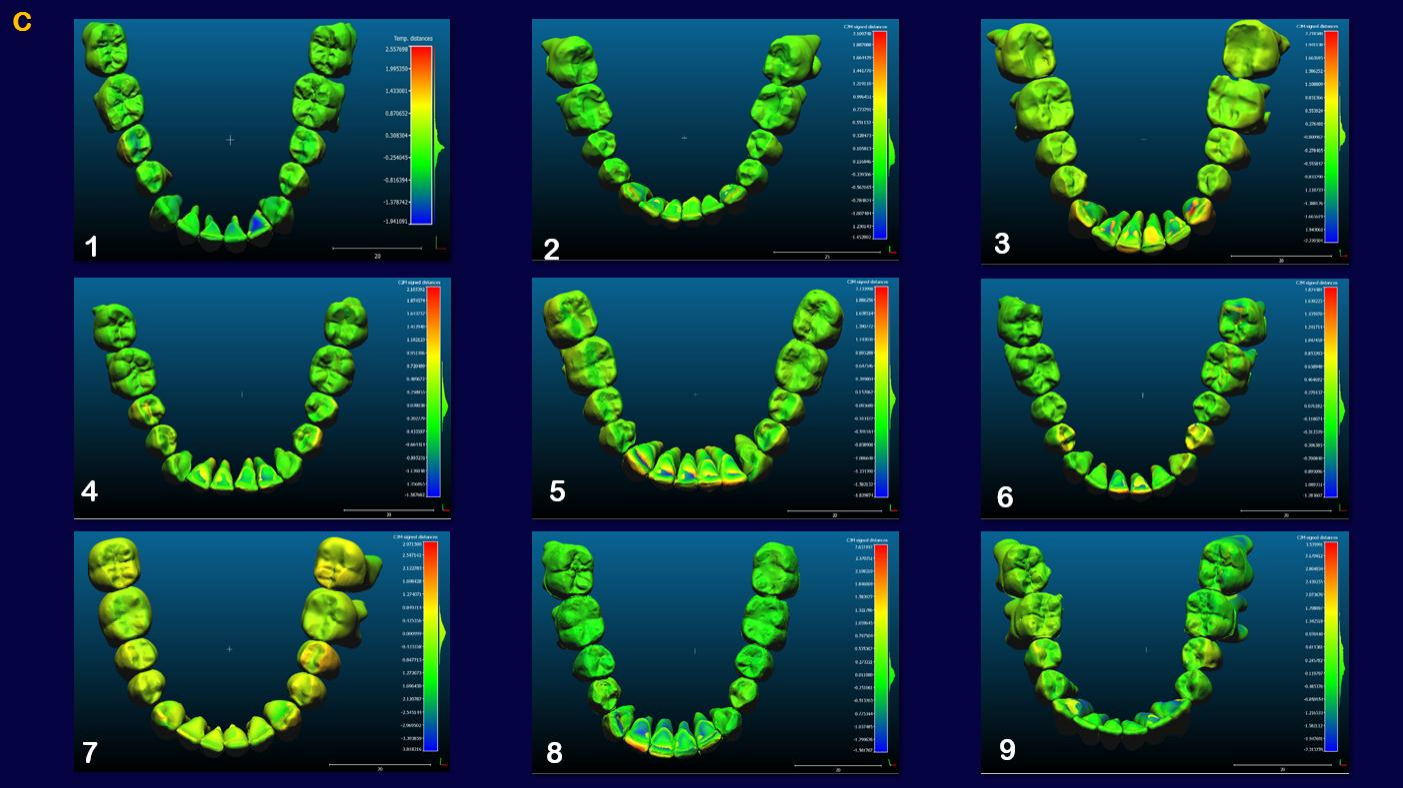
Composite of colour maps showing dental displacements between T0 and T1. Occlusal view.
